# Role of ROCK upregulation in endothelial and smooth muscle vascular functions in diabetic rat aorta

**DOI:** 10.1186/1475-2840-12-51

**Published:** 2013-03-27

**Authors:** Figen Amber Cicek, Hilmi B Kandilci, Belma Turan

**Affiliations:** 1 Department of Biophysics, Faculty of Medicine, Ankara University, Ankara, 06100, Turkey

**Keywords:** Vessel function, Nitric oxide, Diabetes, eNOS, ROCK pathway

## Abstract

**Background:**

The RhoA/ROCK signaling pathway mediates vascular smooth muscle contraction while endogenous NO induces vasodilation through its inhibition. Since myosin light chain phosphatase (MLCP) and eNOS are targeted by RhoA/ROCK upregulation then turn to lead abnormalities in vasculature, we aimed to examine whether less endothelial NO-production and inhibited eNOS together with an upregulation of RhoA/ROCK signaling pathway in thoracic aorta can play an important role in vascular dysfunction under hyperglycemia.

**Methods:**

We used streptozotocin-injected rats, as a model of type 1 diabetes, and their lean controls to investigate the role of ROCK upregulation in the function of toracic aorta by using electrophysiological and biochemical techniques.

**Results:**

The protein level of ROCK isoform ROCK2 was found to be 2.5-fold higher in endothelium-intact aortic rings of the diabetic rats compared to those of the controls while its level in endothelium-denuded rings was similar among these two groups. Phosphorylation level of eNOS in endothelium-intact rings from the diabetics was 50% less compared to that of the control. ROCK inhibitors, either Y27632 or HA1077, induced concentration-dependent relaxation with a marked left-shift in phenylephrine pre-contracted endothelium-intact rings from either diabetics or high glucose incubated controls while pretreatment of these rings with L-NAME abolished this shift, fully. Moreover, phosphorylation levels of both MLCP and MLC in endothelium-denuded rings were markedly higher in the diabetics than the controls.

**Conclusion:**

We demonstrated that diabetes-induced vascular dysfunction can arise due to either inbition of eNOS, thereby less endothelial NO-production, either directly or indirectly, in part, due to an upregulation of ROCK2 by hyperglycemia. Additionally, our data demonstrate that high phosphorylation levels of both MLC and MLCP in endothelium-denuded rings can be due to a less endothelial NO-production dependent ROCK upregulation in the smooth muscle cells under hyperglycemia, as well.

## Introduction

Type 1 diabetes mellitus is characterized by severe hyperglycemia at the whole body as well as several serious functional and structural alterations in the myocardium and vasculature. Endothelial vascular dysfunction plays a key role in the pathogenesis of cardiovascular disorders in diabetes [1,2]. Endothelium controls the tone of the underlying vascular smooth muscle cells through NO-production during physiological and pathological conditions, as well. Previous studies, supporting this statement properly, have shown that there is an association between glucose-induced damage in endothelial cells and decreased NO-bioavailability in the vascular bed [3-5].

The underlying mechanisms of altered contractile responsiveness of vascular smooth muscle cells in diabetes are generally considered to be related to either impaired endothelium-dependent vasorelaxation or to an alteration in the contractility of vascular smooth muscle itself, or both. Accordingly, numerous studies have demonstrated also that diabetes impairs vascular function via inhibition of the endothelium-dependent vasodilatation, accompanied by selective impairment of NO-dependent component of vasodilatation [6]. Indeed, it is known that NO-induced dilatation in arteries is due to an activation of MLCP in a cGMP-dependent manner while MLCP inactivation via RhoA/Rho-kinase (ROCK) pathway antagonizes this Ca^2+^-desensitizing effect [7]. In diabetic preparations, an involment of ROCK-pathway in the mechanical activity of arteries via an increase in active RhoA-kinase level and decreases in both eNOS expression and NO-production by endothelium has been shown already with the previously published data [8,9]. Indeed, in a very recent study, authors determined the role of ROCK isoforms in diabetes-induced vascular endothelial dysfunction and demonstrated that there is a close association between a reduction in endothelial NO-production and upregulation of ROCK while limiting ROCK activity improves vascular function in type 1 diabetic mice [10].

Although several different factors likely play important role in the development of diabetes-associated abnormal vascular reactivity, diabetes-induced altered regulation of smooth muscle cell contraction is particularly through a regulation by the opposing activities of myosin light chain kinase (MLCK) and myosin light chain phosphatase (MLCP) [11]. Indeed, it is well accepted that MLCP can be inhibited via a pathway that involves RhoA/ROCK signaling. This pathway can induce phosphorylation of myosin phosphatase target subunit 1 (MYPT1), a subunit of MLCP, which in turn inhibits MLCP activity [12]. Moreover, Wang et al. [13] showed that two isoforms of ROCK (1 and 2) are expressed in vascular smooth muscle cells, and both inhibit MLCP activity although an isoform-specific role in vascular system although a mechanism of ROCK-mediated regulation of MLC phosphorylation, in part due to eNOS level is not well understood yet. Therefore, we aimed to examine the role of ROCK isoforms and involvement of eNOS (indirectly endothelial NO-production) and MLCP activity as well as their association with hyperglycemia in endothelium-dependent and -independent reactivity of thoracic aorta from diabetic rats.

## Materials and methods

### Induction of type 1 diabetes in rats

All animal care and experimental procedure were performed by following Ankara University ethics guidelines (No: 2007-11-39). Diabetes was induced in 3-month-old male Wistar rats as described previously [14]. Shortly, diabetes was induced by a single intraperitoneal injection (i.p.) of streptozotocin (STZ, 50 mg/kg body weight and dissolved in 0.1 M citrate buffer, pH 4.5) in the rats (200–250 g initial body weight). Control rats received citrate buffer alone. A week after injection of STZ, blood glucose levels were measured and rats with blood glucose at least 3-fold higher than the pre-injection level were used as diabetic rats. All rats had free access to standard rat chow and water, and they were kept for 4 weeks more following the induction of diabetes (totally 5 weeks).

### Histological examination

Histological examination was performed as described previously [14]. For histological examination, aortic rings were fixed in phosphate buffer containing 10% formaldehyde for 2 days. Following sample dehydration through graded alcohol concentrations (50%, 75%, 96% and 100%), the aorta tissue was embedded into paraffin. The embedded samples were sliced at a thickness of 4–6 μm using a Leitz-1512 microtome. Sections were stained with hematoxylin and eosin. Images were obtained by using a Zeiss Axioscope photomicroscope coupled to a charge-coupled device (CCD) camera and analyzed by using color threshold (for collagen and elastin contents).

### Measurement of relaxation responses of aortic preparations

Aortic rings were prepared as described previously [15]. The rings were set at 1-g passive tension and allowed to equilibrate for 60 min, and then first their contractile ability was assessed by exposure to 60 mM KCl.

Endothelium-denuded (endo-) or -intact (endo+) rings were pre-contracted with 1-μM phenylephrine (Phe) in absence or presence of N(G)-nitro-L-arginine methyl ester (L-NAME), and treated with increasing concentrations of a ROCK2 inhibitor Y27632 (10^-9^ - 10^-5^ M). For comparison, we used another inhibitor HA1077 (10^-9^ - 10^-5^ M). Some endo(−) rings were treated with Y27632 before contraction and then relaxed with NO donor sodium nitroprusside, SNP.

To mimic diabetes, aortic rings from control rats were incubated with either only DMEM (5.5-mM glucose) (Biological Industries, Israel) or high glucose (40-mM) containing DMEM for overnight. Following the incubation period, relaxation responses to Y27632 were measured following 1-μM Phe-precontraction.

### Western blot analysis

The frozen aortas were pulverized at liquid nitrogen temperature and then homogenized as described previously [14]. ROCK1/2, eNOS and phospho-eNOS, MLC and phospho-MLC, MYPT1 and phospho-MYPT1 protein levels were determined by using Western blotting. The samples were homogenized in ice cold homogenization buffer (20 mM Tris–HCl at pH 7.4), 150 mM NaCl, 2 mM KCL, 2 mM EDTA, 0.5 mM DTT, 100 mM protease inhibitor cocktail. The homogenate was clarified by centrifugation at 1000 g for 10 min at 4°C, the supernatant was collected and protein concentration was measured with Bradford assay by using BSA as a standard protein. The PVDF membranes were incubated overnight with either ROCK1 or ROCK2 antibodies (1:500, Santa Cruz Biotechnology Inc., Santa Cruz, CA, USA), MYPT1 or phosphospecific MYPT1 (Thr853) antibodies (1:200, Cell Signaling Technology, Inc., Danvers, MA, USA), eNOS or phosphospecific eNOS (Ser 1177), and MLC or phosphospecific MLC antibodies (1:500, Abcam, Cambridge MA, USA) at 4°C, β-actin (1:1000, Santa Cruz Biotechnology Inc., Santa Cruz, CA, USA) antibody was used to control the equal amount loading.

### Chemicals, data analysis and statistics

Unless specified, the reagents used were obtained from Sigma-Aldrich Canada or Fisher Scientific.

Data are presented as mean ± SEM. Concentration-response curves and the negative logarithm of half maximal effective concentration (Log IC_50_) were obtained by using spline fit (curve fitting) with GraphPad Prism (version 5.01). Student t-test or one-way ANOVA followed by tukey-test were used to determine statistical significance (at p < 0.05).

## Results

### Effect of diabetes on general characteristics of rats and thoracic aorta

Five weeks after STZ injection, rats from diabetic group (DM) showed significant increase in water consumption, blood glucose level (432 ± 8.6 mg/dL and 110 ± 4.8 mg/dL in DM *vs.* CON) with marked reduction in body weight (195 ± 6.8 g) compared to those of the controls (241 ± 9.8 g).

**Figure 1:**
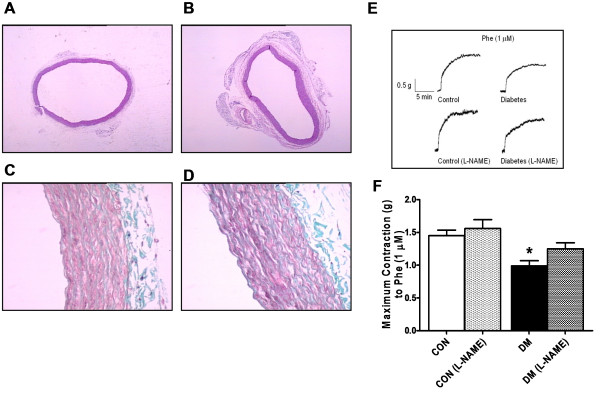
**Effects of diabetes on the structure and contractile function of thoracic aorta.** Light microscopy of endothelium-denuded thoracic aorta. Micrographs of the transverse aortic sections in the control group **(A)**, and the diabetic group **(B)**. Note the unchanged internal diameter of the aorta in the diabetic group compared to that of the control (H. E. X4). Masson’s trichrome stained aorta from the control group **(C)**, and the diabetic group **(D)**. Dark dense part tunica media containing mostly smooth muscle cells and elastin, slightly dense part tunica adventisia containing collagen deposition. Trichrome x40. **(E)** Original traces representing isotonic contraction of phenylephrine (Phe; 1-μM) treated aorta rings from control (top left), L-NAME incubated control (bottom left) and diabetic (top right) and L-NAME incubated diabetic (bottom right) groups. **(F)** The maximum responses to the Phe. Bars represent mean ± SEM; n = 6–8 aortic rings/group. ^*^P < 0.05 *vs.* CON (by using ANOVA with Tukey test).

Significant thickening of smooth muscle bundle in the total wall was observed in the aorta from diabetic rats although there was no significant change in the internal diameter of aortas from the diabetic and the control groups (Figure 1A and B, respectively). Observed basic histological differences in the aorta structure among the control and the diabetic rats are illustrated in Figure 1C and D, respectively. More and better organized smooth muscle cell layers (light pink) in the aorta of the control group were seen between the elastin bundles (dark pink). However, significant thickening of the total aortic wall was observed in the diabetic rats. As published previously [14,16,17], in here, the thickness of the collagen deposition in diabetics was increased as about 3.5-fold compared to that of the control.

### Effect of diabetes on contractile responses to phenylephrine application

We previously demonstrated that STZ-diabetic rats exerted a marked vascular endothelial and contractile dysfunctions including impaired contractile responses to 1-μM Phe or KCl and impaired endothelium-dependent relaxation to either acetylcholine or isoproterenol applications. In here, we first examined the contractile activity in absence or presence of L-NAME in STZ-diabetic rings. Aortic rings isolated from diabetic rats presented a less contractile response to the Phe application compared to that of the control under either absence or presence of L-NAME (Figure 1E). As illustrated in Figure 1F, the maximal responses to Phe-precontraction in the either diabetic group or control group in the presence of L-NAME are not significantly different from those of absence of L-NAME. Our first data with diabetics can provide a first information related with diabetes-induced a contractile vascular function, of which showed, that contractions in response to phenylephrine were not effected from NO-release in diabetic rat aortic rings [14,15,18].

### Effect of diabetes on eNOS protein level in endothelium-intact thoracic aorta

**Figure 2:**
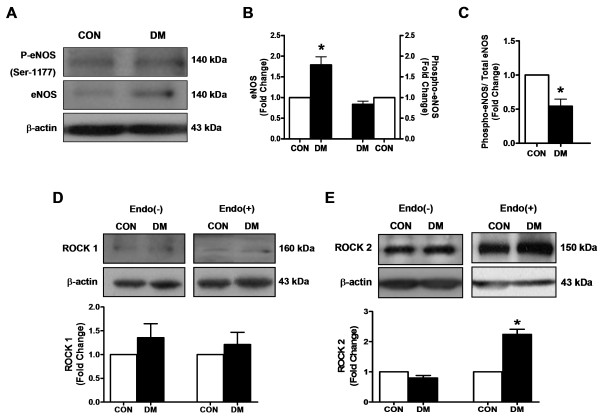
**The phospho/total eNOS and Rho-kinases1/2 (ROCK1/2) protein levels in diabetic rat thoracic aorta. (A)** Blotting data for phospho-eNOS (at Ser 1177), eNOS (140 kDa) and β-actin protein expressions from the control (CON) and the diabetic (DM) rat aorta homogenates. **(B)** Left axis, eNOS and right axis phospho-eNOS was normalized to corresponding β-actin protein band. **(C)** Phospho-eNOS was normalized to corresponding eNOS protein band **(D)** and **(E)** Representative Western Blots of 160 kDa ROCK1, and 150 kDa ROCK2 of endothelium denuded and endothelium intact aorta homogenates from control (CON) and diabetic (DM) rats, respectively. Bar graphs, as mean ± SEM are representing ROCK1 and ROCK2 protein levels which were normalized to β-actin, n = 5 homogenates/group, ^*^P < 0.05 *vs.* CON group (by using student t-test).

In order to demonstrate an involvement of ROCK-pathway via a change in eNOS protein expression level and endothelial NO-production in the aorta from STZ-diabetic rats [8,9], we measured eNOS protein level and its total phosphorylation level in endothelium-intact (endo+) rings (Figure 2A to C). The bars (in Figure 2B and C) are obtained with a normalization of the eNOS protein bands to β-actin protein bands. As can be seen from Figure 2B, the total eNOS protein level in diabetic group was significantly higher than that of the corresponding control group while the phospho-eNOS values were similar among these two groups. Therefore, the phospho-eNOS (at Ser 1177) to total eNOS ratio was found to be decreased as about 50% in the diabetic group compared to that of the control group (Figure 2C). This ratio confirms a decrease level of endothelial NO-production in endo(+) rings from diabetics, which can further induce a functionally inactive endothelial layer in these preparations.

### RhoA through ROCK effectors in isolated thoracic aorta: ROCK1 and ROCK2

In order to demonstrate a role of ROCK isoforms in diabetes-induced vascular dysfunction, we first examined the protein levels of ROCK1 and ROCK2 in both endo(−) and endo(+) aortic rings from both groups of rats (Figure 2C and D, respectively).

Upper parts of Figure 2D and E show the relative level of every substance/β-actin, quantified by using a bioimage analyzer. As shown with bar graphs, the relative protein levels of ROCK1 in both endo(−) and endo(+) aortas did not differ significantly between the diabetic and the control groups. However, the relative protein level of ROCK2 in the endo(+) aortic rings, but not in the endo(−) preparations from the diabetic group was significantly higher than those of the control group. This observation can be associated with the increased ROCK2 protein level which can provide also an important information related with possible correlation between the increased level of ROCK2 expression in the endothelium and a functionally inactive endothelial layer in the aortic rings from STZ-diabetic rats.

### Effect of ROCK inhibition on Phe-precontraction and relaxation responses in the endo(+) and endo(−) aortic rings

**Figure 3:**
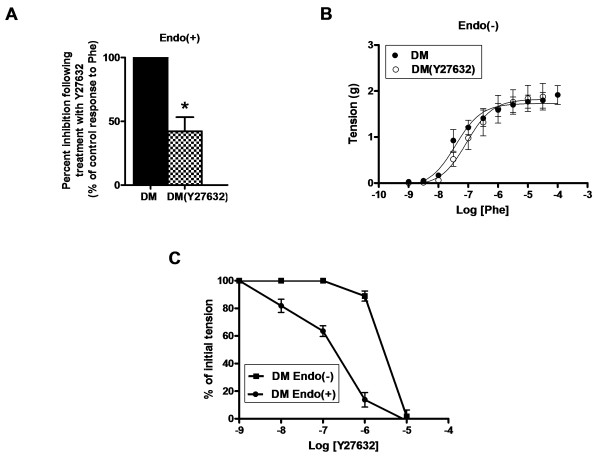
**Effect of ROCK inhibition on Phe-precontraction and relaxation responses in the endo(+) and endo(−) aortic rings. (A)** In the presence of 1-μM Y27632, the contractile response to 1-μM Phe stimulation in diabetic (DM) endo(+) aortic rings was markedly less compared to that of without Y27632. **(B)** The concentration-dependent contractile responses to Phe of diabetic endo- aortic rings, either in the absence or presence of 1-μM Y27632 are overlapped. Data are expressed as mean ± SEM, n = 5-6/group. ^*^P < 0.05 *vs.* without Y27632 samples (by using student t-test). **(C)** DM endo(+) exhibited an increased vasodilator sensitivity to Y27632 compared with endo(−) aortic rings.

To test possible contribution of ROCK signaling pathway into hyperglycemia-induced impaired relaxation responses, we first treated diabetic endo(+) rings with ROCK2 inhibitor, 1-μM Y27632 and then they were contracted with 1-μM Phe. As can be seen from Figure 3A, the contractile response to the Phe in ROCK2 inhibitor-incubated endo(+) diabetic rings is much more smaller that of untreated and control ones (the last data not shown). In a further experiment with endo(−) diabetic aortic rings either treated with Y27632 or not, their concentration-dependent contractile responses to Phe (10^-9^ – 10^-4^ M) are overlapped fully (Figure 3B).

In another set of experiments, either the endo(+) or endo(−) diabetic aortic rings were first contracted with 1-μM Phe and exposed to increasing concentrations of Y27632. The endo(+) rings compared to the endo(−) ones exhibited markedly increased vasodilator sensitivity to Y27632 compared to those of the endo(−) rings (Figure 3C).

### Effect of NO-synthase inhibition on relaxation to ROCK inhibitor in diabetic rat thoracic aorta

**Figure 4:**
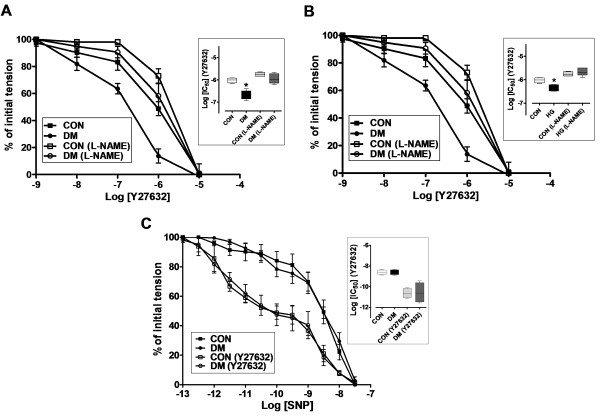
**Effect of NO synthase inhibition with L-NAME on the relaxation response to Y27632 in the precontracted endo(+) aortic rings. (A)** DM endo(+) aortic rings were exhibited an increased vasodilator sensitivity to Y27632 compared with control (CON) aortic rings. In the presence of L-NAME, ROCK inhibition was less effective to both DM and CON groups. Log [IC_50_] values on the relaxation to Y27632 are given in the inset. **(B)** Hyperglycemic (HG) endo(+) aortic rings were also exhibited an increased vasodilator sensitivity to Y27632 compared with CON aortic rings. Their Log [IC_50_] values on the relaxation to Y27632 are given in the inset. **(C)** Effect of ROCK inhibition on relaxation response to SNP after submaximal contraction with 1-μM Phe in endo(−) aortic rings were presented. In the presence of Y27632 an increased and equivalent vasodilatory sensitivity to SNP was observed for both DM and CON aortic samples. The Log [IC_50_] values for the relaxation to SNP are given in the inset. Data are expressed as mean ± SEM, n = 5-6/group. ^*^P < 0.05 *vs.* CON group, by using student’s t test.

To test effect of diabetes on relaxation to ROCK inhibition, the endo(+) rings were first precontracted with 1-μM Phe and then were relaxed with Y27632 (from 10^-9^ to 10^-5^ M) either in absence or presence of L-NAME. As shown in Figure 4A, the aortic rings from diabetics exhibited increased vasodilator sensitivity to Y27632 compared to those of the controls. In presence of L-NAME, ROCK inhibition was significantly less effective in both diabetic and control groups (Figure 4A). The Log [IC_50_] value for the relaxation to 1-μM Y27632 in diabetics (−6.684 ± 0.08) is significantly smaller than that of control (−6.02 ± 0.05), whereas these are found to be similar between these two groups with the presence of L-NAME.

For comparison, we measured relaxation responses with another ROCK inhibitor, HA1077. The relaxation responses to 1-μM HA1077 in diabetics were also exhibited increased vasodilator sensitivity compared to those of the controls (data not shown). The Log [IC_50_] value of the diabetics in the presence of L-NAME (−5.3 ± 0.2) is significantly greater than that of Log [IC_50_] in the absence of L-NAME (−5.9 ± 0.2).

For assessment of diabetes-induced relaxation dysfunction to ROCK inhibition, we performed some experiments with high glucose-incubated rings. Incubation of rings with either 40-mM glucose for overnight exhibited an increased vasodilator sensitivity to 1-μM Y27632 application compared to normal glucose (5.5-mM)-incubated controls. The Log [IC_50_] values are measured as −6.344 ± 0.07 and −6.02 ± 0.05, respectively (Figure 4B). In the presence of L-NAME, similar to the diabetic group, ROCK inhibition was significantly less effective in both high-glucose incubated or normal-glucose-incubated control groups (Figure 4B).

### Effect of Y27632 on endothelial NO-induced relaxation in diabetic rat aorta

To examine the effect of ROCK inhibition on the relaxation response to NO, endothelium-denuded rings were first contracted with 1-μM Phe (in the absence or presence of 1-μM Y27632) and subsequently relaxed with increasing concentrations of sodium nitroprusside, SNP (10^-13^–10^-7.5^ M), a NO donor [19]. Compared with untreated rings, rings treated with 1-μM Y27632 before Phe-precontraction exhibited an increased vasodilator sensitivity to SNP (Figure 4C). This result can suggest that inhibition of ROCK potentiates endothelial NO-induced relaxation. It is important to note that treatment with 1-μM Y27632 did not augment significantly the contractile response in endothelium-denuded rings. These data are in line with a fact of altered relaxation responses to ROCK inhibition in diabetic samples are mostly due to endothelial dysfunction.

### Effect of diabetes on increased phosphorylations of MLCP and MLC in thoracic aorta

**Figure 5:**
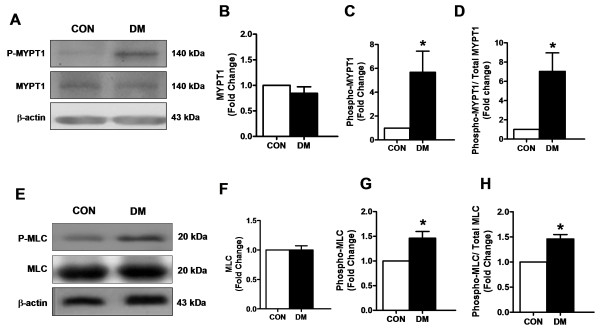
**Phospho/total MYPT1 and Phospho/total MLC expressions in rat thoracic aorta. (A)** Representative Western Blots for phospho-MYPT1, MYPT1 (140 kDa) and β-actin expressions from control (CON) and diabetic (DM) rat aorta homogenates. **(B)** MYPT1 and **(C)** Phospho-MYPT1 normalized to β-actin. **(D)** Phospho-MYPT1 was normalized to corresponding MYPT1 protein band, respectively. **(E)** Representative Western Blots for phospho-MLC, MLC (20 kDa) and β-actin (43 kDa) expressions from CON and DM rat aorta homogenates. **(F)** MLC and **(G)** Phospho-MLC normalized to the β-actin. **(H)** Phospho-MLC was normalized to corresponding MLC protein band, respectively. Bars represent mean ± SEM, n = 5 homogenates/group, ^*^P < 0.05 *vs.* CON group (by using student t-test).

A substrate of ROCK is MLCP, which is physiologically responsible for dephosphorylation of MLC [20]. Therefore, MLCP phosphorylation is a hallmark of ROCK activation [21] which can also phosphorylate MLC directly [22]. To test this pathway in smooth muscle cells from the diabetic rat aortic rings, phosphorylation level of MLCP (phospho-MYPT1) was evaluated. The endo(−) aortic rings from diabetic rats showed no change in the protein level of MYPT1 compared to those of the controls whereas its phosphorylation level was markedly higher in the diabetic preparations (Figure 5A to C). As can be seen from Figure 5D, the phospho-MYPT1 to total MYPT1 ratio of the diabetics is significantly higher than that of the control.

Since inactivation of MLCP is associated with increased phospho-MLC, and a net effect of ROCK activation is associated with increased phospho-MLC, we found that phospho-MLC was found to be markedly higher in diabetics compared to that of control with no change in its protein level. Furthermore, phospho-MLC to total MLC ratio for diabetic endo(−) aortic rings is significantly higher than that of control (Figure 5E to H).

These results indicate that the depressed contractile activity of the aortic rings in diabetics includes, in part, a ROCK inhibition in the smooth muscle cells.

## Discussion

Our data provides new information related with association between important role of RhoA/ROCK pathway via depressed eNOS due to less endothelial NO-production and impaired mechanical activity of aorta under hyperglycemia. The histological data based on the observed aortic stiffness as an indication of thickening of smooth muscle bundle in the total wall structure seem to be closely associated with the observed impaired mechanical activity. Previous studies, similar to ours demonstrated a similar association what is observed in vascular preparations which are strongly supporting the present results also [17,23,24]. Parallel to this fact, we showed that the significant decrease in the total protein level of eNOS, a strong marker of endothelial dysfunction via less endothelial NO-production level [10], is associated with impaired structural and functional properties of endo(+) diabetic aortas. Depressed contractile responses of diabetic endo(+) aortas, with either absence or presence of L-NAME, consistent with diabetes-induced both endothelial and smooth muscle dysfunction, which seems to be, in part, associated with structural alterations also. In addition, although there was no differences between relaxation responses of Phe-precontracted diabetic and control endo(−) rings to SNP, a NO donor, our further data with proteins from contractile machinery provides information related with the contribution of NO-related smooth muscle cell dysfunction onto the endothelium-intact aortic dysfunction. In a supporting study [25], authors demonstrated that RhoA/ROCK signaling pathway could directly suppress NO-production in endothelium [10,26]. These findings are also consistent with early occurence of endothelial dysfunction in the course of diabetic vascular complications [17].

It is known that the vascular endothelium is the first and fundamental part for vascular injury being a common target of hyperglycemia/insulin resistance related risk factors as well [27]. Although many studies were performed with several animal models, there are conflicts related with the vascular responses to contractile agents in the diabetic subjects. Accordingly, some studies suggested increased sensitivity to noradrenaline [28], whereas other studies, including our team’s studies, reported an attenuated contractile response to noradrenaline in the aortas from diabetic animals [14,15,18,29]. These studies mainly stated that the impaired contractile responses to phenylephrine applications could be due to a change in α-adrenergic receptor expression in the vasculature [30]. Furthermore, it has been demonstrated that the aortic preparations from diabetics showed an impaired endothelium-dependent relaxation to acetylcholine [14,15,18,31]. Abnormalities in vessels of diabetic subjects, basically with dysfunction in the microvascular endothelial-barrier, are playing a critical role in the pathogenesis of diabetes-induced vascular dysfunction [27,30,31]. A member of the RhoA family, ROCK is the first and their best-characterized effectors, which are responsible for the modulation of a wide range of cellular functions including contraction. An important support for this fact is given with the data, in which it has been demonstrated an involvement of ROCK-dependent rearrangement of the actin cytoskeleton and changes of cell contractility in the regulation of endothelial-permeability [32,33]. Moreover, much more evidence indicates that expression and activity of eNOS are regulated by the RhoA/ROCK pathway [34,35]. In addition, previously published data also strongly indicated that activation of the RhoA/ROCK pathway is a critical step towards the less endothelial NO-production due to an alteration in the eNOS system in the vasculature from diabetic subjects. Supporting this statement, early and resent some important studies have been shown that ROCK inhibition can induce improvement in diabetes- and hypertension-induced endothelial dysfunction [10,26,36,37]. As can be seen from all previously published data, although we did not examine endothelial NO-production directly, a measurement of decrease phospho-eNOS (at Ser 1177) to total eNOS ratio corresponds to a decreased level of NO-production in the endothelium of diabetic rat aorta. As mentioned by Nunes et al. [38], upregulation of RhoA/Rho-kinase pathway is a key link among many diseases including diabetes [9,39,40]. We measured 2.5-fold higher ROCK isoform ROCK2 protein level in endo(+) aortic rings from the diabetic rats compared to that of the control while its level in endothelium-denuded (endo-) aortic rings was similar among these two groups. Our present data also demonstrated that ROCK inhibitors either Y27632 or HA1077 induced concentration-dependent relaxation with a marked left-shift in the phenylephrine pre-contracted endo(+) rings from either diabetics or high glucose incubated controls while pretreatment of these rings with L-NAME abolished this shift, fully. In addition, we showed that the phosphorylation levels of both MLCP and MLC in endo(−) aortic rings were markedly higher in the diabetics than those of the controls.

There are several targets identified for ROCK signaling pathway including MLC and MLCP in the cardiovascular system [26]. Considering the roles of RhoA-mediating signaling in cellular functions as well as regulation of vascular tone along with inflammation, hyperglycemia, and oxidative stress, the inhibition of this pathway seems to have significant clinical implications [41]. Smooth muscle cell structure and endothelium-dependent/-independent signaling pathways are main factors to control the mechanical activity of vasculature. To demonstrate the hyperglycemia-induced altered factors which contribute to the aortic activity via endothelium, we performed relaxation responses to a NO donor SNP in the endo(−) aortic rings from diabetics and compared them with those of the controls in the absence or presence of ROCK inhibitor Y27632. The similar responses among these two groups can strongly support the role of decreased endothelial NO-production on the upregulation of ROCK signaling pathway in the smooth muscle cells under hyperglycemia. Although we measured significantly high phosphorylation levels of both MYPT1 and MLC in the diabetic endo(−) rings, these higher phosphorylation levels of contractile proteins seem to be not parallel to the results on relaxation responses of diabetic or control endo(−) rings to SNP. As a point of view, since this point seems to be contradictory event in our preparations, the lack of basal phosphorylation effect on SNP-induced relaxations, most probably corresponds to no change in the dynamic range of ROCK activity in the smooth muscle cells from diabetic samples. In addition, our data by measuring the force generation of diabetic endo(+) aortic rings showed that inhibition in 1-μM Y27632 on Phe-precontraction was significantly higher than that of the control, which was similar among these two groups in the presence of L-NAME. This result does further support the fact of a presence of ROCK upregulation signaling in diabetes due to decrease level of NO-production in the endothelium. It is important to note that we observed also similar effect with Y27632 in the relaxation responses to the Phe-precontraction in the high glucose incubated endo(+) rings. These data confirm also strongly a possible role of ROCK activity in the maintenance of agonist-induced force generation and further also support the hypothesis on NO induces the relaxation of vascular smooth muscle cells through the inhibition of RhoA/ROCK–mediated contraction. In here, hyperglycemia seems to be a direct ROCK agonist besides its action via level of NO-production in endothelium, which are also supported with previously published data [8,26,39,42].

**Figure 6:**
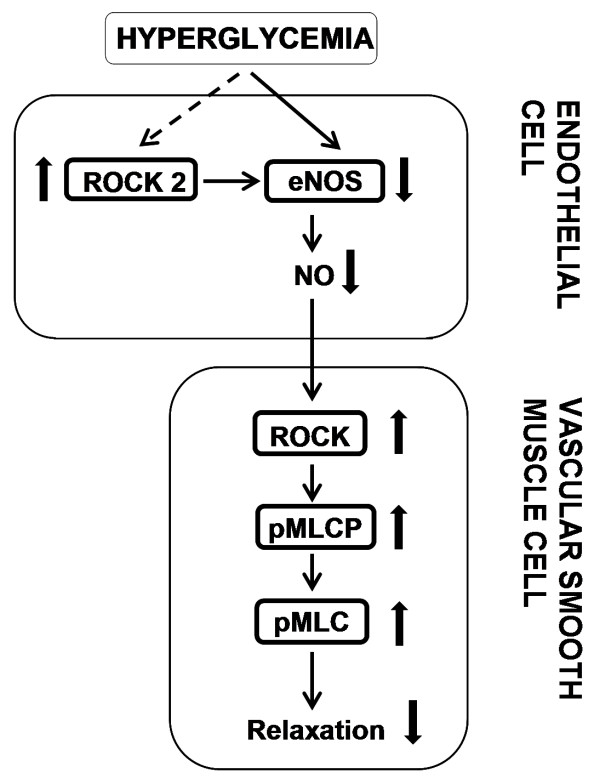
**Proposed mechanism accounting for signal transduction pathways towards the role of RhoA/Rho-kinase signaling in endothelium-dependent both contractile and relaxation activities in thoracic aorta from diabetic rats.** See discussion for details. NO, nitric oxide; eNOS, endothelial nitric oxide synthase; ROCK, RhoA/Rho-kinase, pMLC, phospho-myosin light chain; pMLCP, phospho-MLC phosphatase.

Numerous studies demonstrated an attenuation of endothelium-dependent vasodilation in vasculature under various pathological conditions, often being associated with a decrease in NO bioavailability [41,43]. On the basis of the results presented in here and in the light of previously published data, it is possible to propose a pathway how this signaling is working in our diabetic aortic preparations (Figure 6): It seems hyperglycemia-induced upregulated ROCK2 inhibits eNOS protein expression, which further impairs the NO-release from endothelial cells. Less endothelial NO-production, in turn, can result more activation of ROCK signaling in vascular smooth muscle cells, leading to a decrease in the relaxation responses in the aortic rings. In another word, NO inhibits RhoA/ROCK constrictor activity, which coincides with a decreased level of endothelial NO-production, and/or another possibility of its decreased bioavailability, leading to an increase in RhoA/ROCK constrictor activity. Elevated ROCK activity may then mediate an increased vasoconstrictor sensitivity observed in various animal models with cardiovascular dysfunction [10,26,27,30,31,40].

As summary, the present data demonstrated that diabetes-induced vascular dysfunction can arise due to either inhibition of eNOS, thereby less endothelial NO-production, either directly or indirectly, in part due to an upregulation of ROCK2 by hyperglycemia. Additionally, our data demonstrate that high phosphorylation levels of both MLC and MLCP in endo(−) can be due to a less endothelial NO-production dependent ROCK upregulation in smooth muscle cells as well. Taken together, aortic dysfunction in diabetic subjects seems to be associated with ROCK-mediated signaling pathways via less endothelial NO-production in endothelium which, in turn, results a ROCK-mediated signaling in smooth muscle cells under hyperglycemia. Taking together, our present data provide evidence for a role of ROCK2 in diabetes-induced vascular dysfunction, being a target in both endothelium and smooth muscle cells, and therefore this signaling pathway may present a novel therapeutic means for prevention of diabetes-induced vascular dysfunction.

## Competing interests

The authors declare that they have no competing interests.

## Authors contributions

FAC, Experimental parts, including organ bath and biochemical studies. HBK, Experimental parts including organ bath studies; BT, Planning of the whole study, analysis and writing the manuscript. All authors read and approved the final manuscript.
